# Anterior Lumbar Interbody Fusion With Robotic-Assisted Percutaneous Screw Placement: A Case Report

**DOI:** 10.7759/cureus.22573

**Published:** 2022-02-24

**Authors:** Luke McVeigh, Miracle C Anokwute, Andrew Huh, Nathaniel Blucker, Brandon C Lane

**Affiliations:** 1 Neurological Surgery, Indiana University School of Medicine, Indianapolis, USA

**Keywords:** robotic-assisted fusion, spine robot, alif, percutaneous pedicle screw, anterior lumbar interbody fusion

## Abstract

Recently, there has been an increase in robotic-assisted spine fusion for degenerative spondylosis of the lumbar spine. We present the case of a 60-year-old female with grade 1 spondylolisthesis at L4/5 and L5/S1 who underwent L4-S1 anterior lumbar interbody fusion (ALIF) with percutaneous robotic-assisted pedicle screw fixation. We provide a detailed analysis of the procedure including the speed of robotic screw placement and pitfalls of this surgical approach.

## Introduction

Lumbar spondylolisthesis is an anatomical spinal disorder involving the translation of one vertebral body on another which can cause symptoms of nerve root compression. The management of lumbar spondylolisthesis depends on the etiology, anatomical imaging characteristics, and patient presentation but typically involves lumbar spinal fusion [1-7]. Recently there has been a resurgence in robotic-assisted spinal fusions for a range of spinal disorders resulting in safe and accurate spinal instrumentation [8,9]. We present the case of a 60-year-old female with grade 1 spondylolisthesis where we apply a combination of anterior lumbar interbody fusion (ALIF) with percutaneous robotic-assisted pedicle screw fixation.

## Case presentation

The patient is a 60-year-old female with a past medical history of sarcoidosis and breast cancer treated with surgical resection and chemoradiation that presented with a six-month history of low back pain and dysesthesia radiating down her right posterior thigh and intermittently in the anterior and medial thigh after falling. She denied any left-sided symptoms. She went from ambulating independently to using a walker. She experienced nighttime urinary incontinence due to pain. She failed conservative treatment with lumbar epidural steroid injections, physical therapy, and narcotic pain medications. On examination, she was grade 4+/5 Medical Research Council (MRC) strength in her right hip flexion and knee extension, and 4-/5 right dorsiflexion, plantar flexion, and extensor hallucis longus. She was full strength on the left with normal sensation.

**Figure 1:**
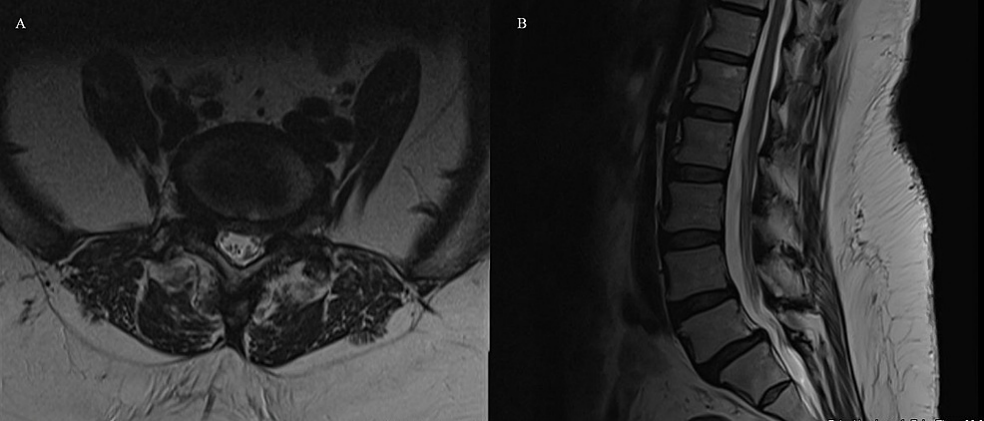
Axial (A) and sagittal (B) T2 weighted MRI of the lumbar spine demonstrating L4/5 and L5/S1 grade 1 spondylolisthesis with foraminal stenosis and minimal central stenosis

CT of the lumbar spine demonstrated no fractures. MRI of the lumbar spine demonstrated grade 1 spondylolisthesis of L4/5 and L5/S1 with nerve root compression in the neuro-foramen, right greater than left. (Figures 1A, 1B). There was no significant central stenosis. Imaging did demonstrate significant facet arthropathy bilaterally. Upright and flexion-extension x-rays of the lumbar spine re-demonstrated the grade 1 spondylolisthesis, but also showed micro-motion instability at L4/5 and L5/S1. Standing scoliosis x-rays demonstrated minimal levoscoliosis of the lumbar spine. Body habitus precluded spino-pelvic measurements including pelvic incidence, pelvic tilt, and sacral slope.

Given our patient’s unilateral symptomatology, grade 1 spondylolisthesis, and no significant central stenosis, she was offered an L4-5 and L5-S1 ALIF with percutaneous robotic-assisted posterior percutaneous lumbar spinal fusion. A vascular surgeon was used for abdominal access.

Operative procedure

Both the anterior approach and posterior fusion were performed under general anesthesia. The patient was supine for the anterior approach. We utilized a vascular surgeon to gain retroperitoneal access to the anterior spine. A bent spinal needle was placed into the L4/5 and L5/S1 intervertebral disc space and fluoroscopy was utilized to ensure the placement was midline and at the correct lumbar level. The spinal needle was removed and the midline was marked at L4/5 and L5/S1.

We began the ALIF with an annulotomy at L5/S1 along the implant sizer. A discectomy was performed to the level of the posterior longitudinal ligament (PLL). Fluoroscopy was utilized to gauge the depth of the discectomy. Prior to placement of the interbody, the retractor over the iliac vein dislodged, requiring repositioning. A minor injury to the common iliac vein occurred and was repaired under direct visualization by the vascular surgeon. Under fluoroscopic guidance, a 32x23x12mm 12º cage packed with allograft was placed into the L5/S1 disc space. This was secured with a 25-mm screw into the inferior endplate of L5. Fluoroscopy was utilized to ensure the appropriate placement of both the graft and the screw. At L4/5 we placed a 32x23x14mm 8º cage with allograft. This was anchored into the L4 inferior endplate with a 25-mm screw. Lateral and anterior-posterior (AP) fluoroscopic images were obtained and demonstrated good alignment of the grafts. The vascular surgeon then performed closure of the abdominal incision. It took one hour and 15 minutes from disc incision to graft placement for the L5/S1 level and 28 minutes from disc incision to graft placement at the L4/5 level.

Robotic-assisted percutaneous posterior lumbar spinal fusion

The patient was then positioned prone on a Jackson table with her arms extended towards her head. Using fluoroscopy, we identified the posterior superior iliac spine (PSIS) to mark the location for Schanz pin placement for robot mounting. The patient was then prepped and draped in the usual sterile fashion. The Mazor robot (®Medtronic) was mounted and draped sterilely. A stab incision was made at the marked location along the PSIS and the Schanz pin was placed via battery-powered drill into the right PSIS. The robotic navigation was performed in sequence with three-define spatial orientation and O-arm intraoperative CT imaging for stealth navigation. It took 27 minutes to mount the robot and drape the patient. After completion of stealth CT, software was utilized to pre-plan percutaneous screw trajectories at L4, L5, and S1 bilaterally then uploaded to the robot for guidance. Screw trajectory planning took 17 minutes (Table 1).

**Table 1:** Surgical Time With Robotics

Surgical Time with Robotics	
Procedure	Time (Hrs: mins)
ALIF Access to L5/S1 Disc Annulotomy	5:06
L5/S1 annulotomy to graft placement	1:15
L4/5 annulotomy to graft placement	0:28
Sterile robot draping to O-arm spine	0:27
Screw Planning	0:17
Bilateral L4 Screw Placement	0:08
Bilateral L5 Screw Placement	0:12
Bilateral S1 Screw Placement	0:04

The robot transitioned to the preplanned trajectories for each screw, and these entry points were marked on the patient’s skin. An incision was planned to allow for optimal accommodation of all three trajectories on each side. After incising down to the level of the fascia, the robot then transitioned to the planned trajectory for the left L4 screw. The fascia was incised down to the level of the screw entry point with a large specialized scalpel through the robotic guide arm. The drill guide was advanced through the robotic arm down to the screw entry point. The drill guide was anchored to the cortex of the entry point by a mallet. This allowed for two-point fixation of the drill guide, one to the robotic arm, and the other directly to the cortex. The drill was then advanced down the drill guide and a pilot hole was initiated in the pedicle with stealth navigation. We subsequently lengthened and expanded our pilot hole with an awl-tip-tap to the length of the pre-planned screw.

The awl-tip-tap was removed and a 6.5x40mm screw was placed. This process was repeated in an iterative fashion with 6.5x40mm screws placed at L4, L5, and S1 pedicles. We encountered concern for navigation accuracy while placing the right L5 pedicle screw. The demonstrated trajectory was medial along the pedicle and encroaching on the central canal. The drill was removed, the robotic arm elevated and stealth accuracy rechecked. The stealth was found to be accurate. The robotic arm was navigated closer to the patient to minimize torque from the weight of the drill. The screw was placed as above. Screw placement time at L4 was eight minutes between screws, 12 minutes between screws at L5, and four minutes between screws at S1 (Table 1).

We then obtained an intraoperative O-arm stealth CT to evaluate screw placement accuracy. This demonstrated a right L5 medial pedicle wall breach. The right L5 screw was then subsequently removed and not replaced. The rods were secured to the pedicle screw heads utilizing set screws. The wound was washed with saline irrigation and 1g of Vancomycin powder. The Schanz pin was removed and the wound was closed with monocryl suture.

**Figure 2:**
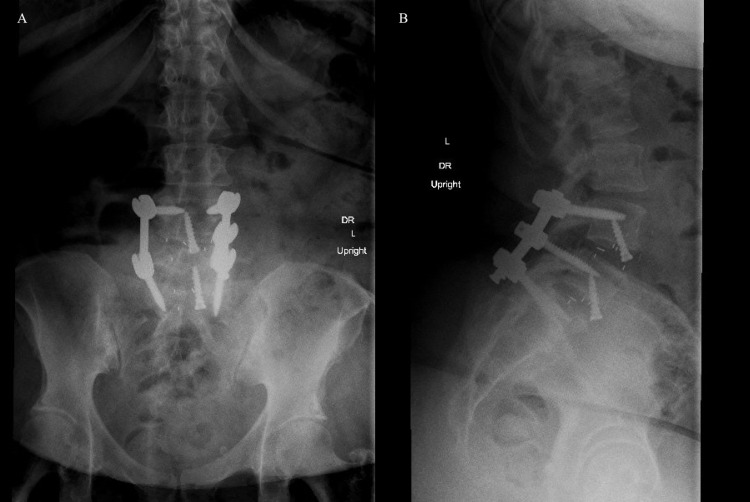
Postoperative upright AP (A) and lateral (B) x-rays AP - anteroposterior

Postoperatively, there were no physical signs of deep venous thrombosis or signs of hematoma on neurovascular examinations. The patient was able to ambulate with a walker and was discharged to an acute rehab facility. She had no left-sided weakness, her back and radicular pain had significantly improved, and she was 5/5 MRC strength in right hip flexion, knee extension, and 4+/5 dorsiflexion, plantarflexion, and extensor hallucis longus at four weeks and at three months follow up. She was ambulating without a walker. Upright x-rays demonstrated good alignment without hardware failure (Figures 2A, 2B).

## Discussion

Indications for ALIF

Lumbar interbody fusion (LIF) can be completed via multiple different approaches including posterior (PLIF), transforaminal (TLIF), oblique (OLIF), lateral (LLIF), and anterior (ALIF). While there are many different approaches, there is currently no consensus on which approach is superior when evaluating fusion rates and clinical outcomes [1]. Choice of approach is highly dependent on specific pathology, surgeon preference, and indication for LIF. Indications for ALIF include degenerative disc disease after failed conservative management, discogenic disease, revision of posterior fusion, minor scoliosis, and low-grade spondylolisthesis [2]. For cases involving spondylolisthesis withly central stenosis and bilateral symptoms, PLIF is often preferable as it allows for decompression while maintaining support via posterior instrumentation and leads to a high rate of fusion [3]. ALIF is particularly effective for pathologies involving L4/L5 and L5/S1 as these spinal levels are located at or below the bifurcation of the abdominal aorta. ALIF can be advantageous in these cases as it allows for increased access to the intervertebral disc space. This can allow for more complete disc removal and accommodation of larger implants [1]. For our patient, multiple factors affected our decision to utilize an ALIF approach. First, she presented with unilateral symptoms. Secondly, imaging demonstrated only a low-grade spondylolisthesis at L4/L5 and L5/S1. And lastly, she did not have any central stenosis. In this instance, we felt that stabilization of her spondylolistheses and indirect foraminal decompression with anterior interbody graft would allow for excellent symptom treatment without disrupting the posterior elements.

While ALIF was determined to be the best approach for this patient, ALIF does have its shortcomings. One of the risks of ALIF is the potential for vascular injury due to the required retraction of the iliac vessels in order to access the disc space [4]. This particular complication did occur in our patient as there was bleeding from the common iliac vein. This should be controlled quickly and efficiently to prevent the risk of hematoma or deep venous thrombosis and the patient should be monitored with serial neurovascular examinations in the postoperative period. However, this highlights the risk of vascular injury and its potentially serious consequences.

Role of indirect decompression for lumbar radiculopathy

Lumbar radiculopathy can result from a multitude of etiologies causing compression of the lumbar foramen. In this particular case, grade 1 spondylolisthesis at L4/5 and L5/S1 led to nerve root compression in the neuro-foramen, greater on the right than on the left. While direct decompression utilizes the complete removal of compressive tissues, indirect decompression involves an expansion of neural corridors without directly manipulating the compressing tissues [5]. Interbody fusion is one method of providing indirect decompression of spinal nerves. ALIF in particular is an effective method of indirect decompression for lumbar radiculopathy as it allows for a larger area of access to the intervertebral space and utilization of comparatively larger grafts with greater expansion of neural corridors [5]. This graft placement via ALIF results in significant foraminal decompression [6,7]. Additionally, reduction of spondylolisthesis and motion stabilization facilitate the minimization of micro-motion instability.

Percutaneous robotic assistance vs fluoroscopic free hand screw placement vs Stealth navigation

For the posterior aspect of the lumbar spinal fusion, we chose to utilize a percutaneous robot-assisted approach rather than using traditional fluoroscopic free-hand screw placement. When choosing between fluoroscopic free-hand screw placement vs robotic assistance, there are multiple reported benefits of robotic assistance. A primary advantage of robotic assistance is a reduction in intraoperative complications. Multiple groups have reported a reduction in complications with robotic-assisted screw placement versus fluoro-guided minimally invasive screw placement, some of which report up to a five-fold reduction in complications [8,9]. A primary contribution to the reduced complication rate is the increased accuracy with robotic assistance. For example, Kantelhardt et al. [9] reported accurate screw placement in 94.5% of robot-assisted cases versus 91.4% of conventionally free-hand cases [9]. Similarly, Schalto et al. [10] report perfectly placed screw trajectories in 83.6% of patients treated with robotic assistance versus 79.8% of patients with fluoroscopy-guided screw placement [10]. In our case, one of six (17%) screws (right L5) were placed inaccurately with a medial breach of the pedicle wall even with robotic assistance. It should be noted, neither robot-assisted nor fluoroscopically guided free-hand pedicle screw placement produces perfect accuracy. There also remains a significant learning curve associated with using robotic assistance. A positive correlation has been reported between surgeon robotic experience and the accuracy of screw placement [11]. Significant rapid improvement in accuracy with robot-assisted techniques have been reported in the first six months of robot use as well as a significant increase (82% to 93%) in the first 30 cases [11,12].

In addition to increased accuracy, percutaneous robot-assisted techniques are associated with decreased surgical time and more rapid screw placement. Multiple studies have reported decreased operative time with the use of percutaneous robotic-assisted technique versus fluoroscopic-guided free hand screw placement [9,10,13,14]. In our experience with this case, we saw a reduction in our operative time while utilizing the robot-assisted technique (Table 1). While there was added time for robot draping and screw planning (27 and 17 minutes, respectively), the time required for screw placement was four minutes per screw (eight minutes per bilateral screw placement). This average time for screw placement includes the additional time required to confirm alignment while placing the right L5 screw. In total, including set up time, the robot-assisted portion of this procedure took 68 minutes to place six screws. These times compare favorably with the reported operative time for screw placement by conventional freehand techniques [10,14,15].

Lastly, robotic-assisted techniques allow for minimally invasive approaches. The use of percutaneous screw placement is associated with shorter hospital stays and less post-operative pain than conventional freehand screw placement due to reduced muscle dissection and retraction [8,9]. At four weeks post-op, our patient has shown promising results with significantly improved back pain and radicular pain as well as strength improvement. Our patient is now able to walk short distances unassisted, a major improvement from her pre-op functional status.

## Conclusions

We demonstrate a unique approach utilizing a 360°, minimally invasive, robotic-assisted spinal fusion in combination with an ALIF for degenerative lumbar spondylolisthesis leading to improved symptoms of lumbar radiculopathy. Robotic-assisted spine surgery can be effective however pitfalls including screw misplacement can still occur.
